# Digital Support Interventions for the Self-Management of Low Back Pain: A Systematic Review

**DOI:** 10.2196/jmir.7290

**Published:** 2017-05-21

**Authors:** Barbara I Nicholl, Louise F Sandal, Mette J Stochkendahl, Marianne McCallum, Nithya Suresh, Ottar Vasseljen, Jan Hartvigsen, Paul J Mork, Per Kjaer, Karen Søgaard, Frances S Mair

**Affiliations:** ^1^ General Practice & Primary Care Institute of Health & Wellbeing University of Glasgow Glasgow United Kingdom; ^2^ Department of Sports Science and Clinical Biomechanics University of Southern Denmark Odense Denmark; ^3^ Nordic Institute of Chiropractic and Clinical Biomechanics Odense Denmark; ^4^ Department of Public Health and Nursing Faculty of Medicine and Health Sciences Norwegian University of Science and Technology (NTNU) Trondheim Norway

**Keywords:** low back pain, self-management, mHealth, eHealth

## Abstract

**Background:**

Low back pain (LBP) is a common cause of disability and is ranked as the most burdensome health condition globally. Self-management, including components on increased knowledge, monitoring of symptoms, and physical activity, are consistently recommended in clinical guidelines as cost-effective strategies for LBP management and there is increasing interest in the potential role of digital health.

**Objective:**

The study aimed to synthesize and critically appraise published evidence concerning the use of interactive digital interventions to support self-management of LBP. The following specific questions were examined: (1) What are the key components of digital self-management interventions for LBP, including theoretical underpinnings? (2) What outcome measures have been used in randomized trials of digital self-management interventions in LBP and what effect, if any, did the intervention have on these? and (3) What specific characteristics or components, if any, of interventions appear to be associated with beneficial outcomes?

**Methods:**

Bibliographic databases searched from 2000 to March 2016 included Medline, Embase, CINAHL, PsycINFO, Cochrane Library, DoPHER and TRoPHI, Social Science Citation Index, and Science Citation Index. Reference and citation searching was also undertaken. Search strategy combined the following concepts: (1) back pain, (2) digital intervention, and (3) self-management. Only randomized controlled trial (RCT) protocols or completed RCTs involving adults with LBP published in peer-reviewed journals were included. Two reviewers independently screened titles and abstracts, full-text articles, extracted data, and assessed risk of bias using Cochrane risk of bias tool. An independent third reviewer adjudicated on disagreements. Data were synthesized narratively.

**Results:**

Of the total 7014 references identified, 11 were included, describing 9 studies: 6 completed RCTs and 3 protocols for future RCTs. The completed RCTs included a total of 2706 participants (range of 114-1343 participants per study) and varied considerably in the nature and delivery of the interventions, the duration/definition of LBP, the outcomes measured, and the effectiveness of the interventions. Participants were generally white, middle aged, and in 5 of 6 RCT reports, the majority were female and most reported educational level as time at college or higher. Only one study reported between-group differences in favor of the digital intervention. There was considerable variation in the extent of reporting the characteristics, components, and theories underpinning each intervention. None of the studies showed evidence of harm.

**Conclusions:**

The literature is extremely heterogeneous, making it difficult to understand what might work best, for whom, and in what circumstances. Participants were predominantly female, white, well educated, and middle aged, and thus the wider applicability of digital self-management interventions remains uncertain. No information on cost-effectiveness was reported. The evidence base for interactive digital interventions to support patient self-management of LBP remains weak.

## Introduction

The point prevalence of low back pain (LBP) is estimated to be 12% and one-month prevalence 23% across the globe [1]. The Global Burden of Disease study reported that LBP is the greatest contributor to disability in 12 of 21 world regions studied [2]. When considering years lived with disability, LBP is one of the leading causes of burden worldwide out of 291 conditions considered [2,3]. It is among the most common causes of long-term work absence and has a major impact on productivity at work [4,5]. Annual costs of LBP have been estimated to be approximately £10.7 billion for indirect factors in the United Kingdom [6,7] and up to US $200 billion in the United States [8], including workplace productivity costs; personal costs include a reduction in everyday functioning and quality of life [9].

Optimizing treatment strategies that are cost-effective, safe, and easy to administer for individuals with LBP is essential. Self-management is consistently recommended in international guidelines on the management of LBP [10,11]. Self-management focuses on the patient’s ability to manage their own condition rather than treatment being based within the health care system or centered on a health care professional. The aim is to restore autonomy to the patient and include educational, or learning, components to position the patient at the center of their own management process and to help them acquire and maintain competencies to enable them to efficiently manage their condition [12].

A systematic review of the effectiveness of the self-management of LBP published in 2012 reports moderate quality evidence that self-management interventions have small, but clinically relevant, effects on reducing pain and disability for people with LBP when compared with minimal interventions [13]. The content and mode of delivery varied across the studies included, from receiving written information, attending face-to-face educational programs, functional movement training programs to information from websites [13].

Digital interventions (ie, interventions accessed via computer, mobile phone, or other handheld devices, including Web-based, desktop computer programs, or apps), providing self-management information have been proposed as a promising mode of delivery for self-management interventions. In a Cochrane Review from 2005, the use of such digital interventions was evaluated in people with chronic diseases and found to have a significant positive effect on knowledge, social support, and clinical outcomes in conditions such as diabetes and obesity [14]. Digital interventions have also been shown to effectively improve chronic pain, including chronic LBP, when compared with control groups (no care, waiting list, placebo, or care as usual) [15]. Providing supported self-management through digital platforms may enable individuals with LBP to better manage their symptoms. Garg et al [16] identified 9 randomized controlled trials (RCTs) for a systematic review of Web-based interventions to support individuals with LBP; included studies were grouped into cognitive behavioral therapy (CBT), a dialogue-based therapy that has been shown to have some efficacy for individuals with LBP [17], or knowledge improvement approaches with an interactive component [16]. Web-based methods were found to be useful, particularly CBT and those that offered an interactive support component; however, there was caution placed on the external validity of all studies included. Consequently, it appears that digital interventions hold potential in supporting the self-management of LBP but not enough is known about their content, delivery, and benefits, if any, or whether these interventions can be expected to be an improvement on traditional self-management approaches. Although it appears that the majority of digital interventions in this area have targeted individuals with chronic LBP (LBP for 3 months or longer), there is little known about the sociodemographic characteristics of individuals with LBP who are either targeted or who subsequently engage with such interventions.

The purpose of this systematic review was to synthesize published evidence concerning the characteristics, components, and effects of interactive digital interventions to support patient self-management of LBP. More specifically, the review aimed to address the following questions:

What are the key characteristics and components of digital self-management interventions for LBP, including theoretical underpinnings?

What outcome measures have been used in randomized trials of digital self-management interventions in LBP and what effect, if any, did the intervention have on these?

What specific characteristics or components, if any, of interventions appear to be associated with beneficial outcomes?

**Inclusion and exclusion criteria.**

**Inclusion criteria**

Participants: adults (18 years or above) with nonspecific LBP

Digital intervention:

- Any intervention accessed through a computer (work or home), mobile phone, or hand-held device, and included Web-based or desktop computer programs or apps that provided self-management information or material, which is in keeping with previous reviews in this sphere [19].
- Element of interaction between the user and digital interface: interaction was defined as patients entering data into the program or app, either by entering personal data or making choices that alter the pathways in the program and produce feedback in response to the patients’ inputted data or choices.
- Interactive component as an add-on to face to-face health professional contact (eg, regularly seeing doctor but reporting pain levels electronically and receiving automated messages advising on physical activity level between visits).

Control group: usual care or digital noninteractive or nondigital self-management interventions for LBP

Study design: published randomized controlled trials (RCTs) or protocols for RCTs from peer-reviewed journals

Language: studies published in English, Danish, or Norwegian

**Exclusion criteria**

Digital intervention:

- Studies that only involved sending information to a remotely located health professional and receiving advice directly from the health professional.

Study design: all non-RCT reports and protocols

## Methods

### Study Design

The systematic literature review followed an *a priori* defined protocol as registered in PROSPERO (reference number 42016037954) and reporting is consistent with the Preferred Reporting Items for Systematic Reviews and Meta-Analyses (PRISMA) statement [18]. Inclusion and exclusion criteria are outlined in Textbox 1.

### Information Sources and Search Strategy

A systematic search of the following databases was undertaken: Cumulative Index to Nursing and Allied Health Literature (CINAHL), Cochrane Database of Systematic Reviews (CDSR), Cochrane Central Register of Controlled Trials (CENTRAL), Cochrane Library (including Database of Abstracts of Reviews of Effects [DARE] and Health Technology Assessment [HTA] databases), Database of Promoting Health Effectiveness Reviews (DoPHER), Embase, MEDLINE, PsycINFO, Trials Register of Promoting Health Interventions (TRoPHI) and Web of Science (Social Science Citation and Science Citation Index). All databases were searched from 2000 until March 2016. Reference and citation searching were also undertaken. The searches were performed by an experienced Librarian at the Norwegian University of Science and Technology (NTNU). The search strategy included subject indexing terms and free-text terms for title, abstract, and keyword searching. The search terms were grouped into 3 concepts: (1) back pain, (2) digital interventions, and (3) self-management. The search terms were selected with reference to previous systematic reviews of interactive digital interventions for hypertension [19,20] and asthma [21,22] and after discussion with the review team. The full version of the search terms used, including specifications on use of title, keywords, or abstract screening, is documented for the example of MEDLINE in Multimedia Appendix 1.

### Study Selection

All identified citations from the searched databases were uploaded to Distiller software (Evidence Partners). An integrated duplication detection tool was used to identify duplicates. All suggested duplicate pairs were screened for correctness by one reviewer (LS). Title and abstract screening was performed for each article by two independent reviewers from four (LS, BN, MM, NS). Disagreement between the two reviewers resulted in inclusion of the citation to full-text screening. Full-text screening was similarly performed by two independent reviewers from four (LS, BN, MM, NS), assessing the eligibility of the citation. Any disagreement was resolved through discussion mediated by a third reviewer (PJM).

### Data Collection

Similar to the study selection process, data extraction was performed independently by two of four reviewers (LS, BN, MM, NS) using the Distiller software. Discrepancies in data extracted were considered by LS by revisiting the original paper to adjudicate on appropriateness and discussed and finalized with BN where required. Data were systematically extracted on study settings (country, inclusion and exclusion criteria, recruitment and participation numbers); study population (baseline characteristics such as age, gender, ethnicity, duration of symptoms, comorbidities); description of the intervention (details on the key components, characteristics, and underlying theoretical concepts); and outcome measures (time-points for outcome assessment, choice of primary outcomes, included secondary outcomes and effects, if any, noted as well as attrition rates, where available).

### Outcome Measures

Our primary and secondary outcomes of particular interest are outlined in Textbox 2. These outcomes were a priori defined as of interest, however all outcomes reported were included in the data synthesis. For this review, pain-related disability was of special interest, as it measures a construct of the physical functioning domain, which has been recommended as a core domain in LBP research by several authors and guidelines [23-25].

### Quality Appraisal

The methodological quality of all included studies was assessed using the Cochrane Collaboration tool for assessing risk of bias in randomized trials [26]. Two reviewers independently assessed selection bias (allocation concealment and randomization procedure); blinding of participants, personnel, and outcomes assessors; completeness of data; selective outcome reporting; and other potential biases. Any disagreements were resolved through discussion by the two independent reviewers (BN, PK). Papers were not excluded from the study on the basis of quality.

### Data Synthesis

The study population, intervention components, outcomes, and characteristics of the included studies were narratively described. In our protocol we stated that we would conduct a meta-analysis if included studies were sufficiently homogeneous; however, due to the heterogeneity of identified studies, meta-analysis was not possible. Quantitative results from all outcomes reported in the completed RCT studies were described as either favoring the intervention group, no difference between groups, or favoring the control group. The outcomes reported in Textbox 2 were used as a basis to structure the results for research question 2. Included protocols for future RCTs were used to consider intervention components, characteristics, and outcome measures, but were not included in synthesis of intervention effects.

## Results

### Study Selection

**Figure 1:**
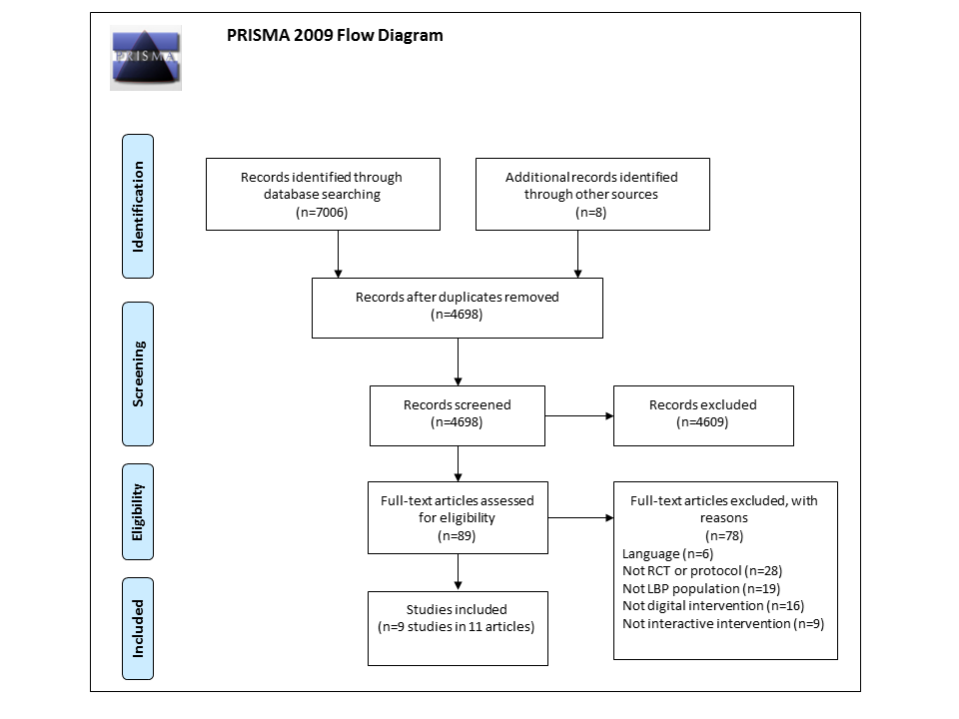
PRISMA (Preferred Reporting Items for Systematic Reviews and Meta-Analyses) flow diagram illustrating the screening process of papers.

We identified a total of 7014 citations, including 8 from searching reference lists of included studies. From these, 2316 were excluded as duplicates, and thus a total of 4698 titles were screened, resulting in the screening of 729 abstracts and 89 full-text papers. A total of 11 references concerning 9 different studies that described 5 RCT study protocols and 6 RCT reports met the inclusion criteria [27-37]. The PRISMA flow diagram demonstrating the screening process is illustrated in Figure 1 (adapted from Moher et al [18]).

**Outcome measures of interest.**

**Primary outcome**

Details of outcome measures used to determine the effects of interventions for self-management of LBP pain-related disability

**Secondary outcomes**

- Pain intensity
- Quality of life
- Depression
- Fear avoidance
- Pain catastrophizing
- Physical activity
- Medication use
- Health care utilization (eg, primary and secondary care visits, emergency department visits)
- Health care costs
- Knowledge of LBP
- Markers of self-care
- Self-efficacy

### Description of Included Studies

Four of the 9 separate studies were undertaken in the United States [28,29,32,33], 2 in Germany [35,37], one in the United Kingdom [31], one in Australia [27], and one in Spain [36]. The studies were published between 2010 and 2016. The 6 completed RCT reports included a total of 2706 participants, with a range of 114-1343 participants per study (Table 1).

### Study Population

The characteristics of the study population in each of the studies are described in Table 1. There was considerable variation between studies in the duration of LBP symptoms, content and delivery of the interventions, and the measured outcomes. In 6 studies, LBP was defined by participant self-report [27-29,32,35,37] and in 3 studies by general practitioner evaluations [31,36] or diagnosis codes from medical records [33]. Seven studies included participants with pain for more than 3 months [27-29,32,33,36,37]. Only one study included participants with current LBP at the time of screening (or within the past 2 weeks) [31], while Simon et al [35] included only acute LBP participants, defined as participants who had experienced pain for less than 3 months. The included populations had a mean age ranging from 42.5 to 52.7 years; one study did not report the age of the population, except to say they were 18-65 years [32], a further study also had an upper age limit of 65 years [36]. All the remaining 7 studies did not report any upper limit in their inclusion criteria, yet only one study reported the age range of participants, 18-79 years [29]. In 5 [28,29,32,35,37] of the 6 RCT reports, the majority of the participants were female (58%-83%). The 6th study, which was conducted within the American Department of Veterans Affairs, included only 11% females in the intervention group and 14% in the control group [33]. Included participants were generally Caucasian (74%-87%) and the majority (42%-75%) reported educational levels as time spent at college or higher.

**Table 1:** Included studies and population characteristics.

Study	Definition of LBP^a^	Number	Age, mean (SD)^b^	Sex (%)	Ethnicity (%)	Comorbid (%)	SES (%)^c^
Chiauzzi et al [29] United States	LBP ≥10 days/month for at least 3 consecutive months	N=209 I^d^=104 C^e^=105	I=47.3 (12.2) C=45.0 (11.7)	I=F^f^ (67) C=F (68)	White I=85 C=87	N/R^g^	Education I=72 C=77 report partial college or associates degree or higher Income, I=79 C=84 report ≥US $25,000/year
Simon et al [35] Germany	Acute LBP (<3 months)	N=1343 I=691 C=652	I=45.8 (12.7)^h^ C=45.3 (13.0)^h^	F (82)^h^	N/R	N/R	Education 60% “high” education level^h^
Carpenter et al [28] United States	Noncancer LBP ≥6 months	N=141 I=70 C=71	42.5 (10.3)	F (83)	White (77)	N/R	Education 54% ≤2 years college
Krein et al [33] Krein et al [34]^i^ United States	Patients with ≥2 outpatient encounters within the past 12 months with a diagnosis of back pain with no neurologic findings (ICD­-9-­CM codes 724.2, 724.5, 846.0­-846.9)	N=229^h^ I=111 C=118 130/group^j^	I=51.2 (12.5) C=51.9 (12.8)	I=F (11%) C=F (14)	White I=74 C=86	N/R	Education I=72 C=75 reported some college or higher Income, I=82 C=87 ≥US $10,000/year
Irvine et al [32] United States	Nonspecific LBP within the past 3 months	N=398 I=199 C=199	N/R	I=F (58%) C=F (63)	White I=76 C=82	N/R	Education I=87 C=94 report some college or higher Income, I=89 C=94 ≥US $ 20,000/year
Weymann et al [37] Dirmaier et al [30]^i^ Germany	Chronic LBP: pain almost every day for >12 weeks	N=368^h^ Randomized I=190 C=188 207/group^j^	I=52.2 (13.1)^h^ C=52.7 (13.0)^h^	I=F, 162 (59)^h^ C=F, 162 (59)^h^	N/R	N/R	Education^h^ I=53 C=51 reported >10 years of education
Geraghty et al [31]^i^ United Kingdom	LBP in the past 3 months recorded in General Practitioner records and current LBP (or within the past 2 weeks) at the time of screening	20-30/group	-	-	-	-	-
Valenzuela-Pascual et al [36]^i^ Spain	Chronic LBP >6 months, confirmed by clinician	29/group	-	-	-	-	-
Amorim et al [27]^i^ Australia	Chronic LBP persisting for >12 weeks but without radicular symptoms	34/group	-	-	-	-	-

^a^LBP: low back pain.

^b^SD: standard deviation.

^c^SES: socioeconomic status.

^d^I: intervention group.

^e^C: control group.

^f^F: female.

^g^N/R: not reported.

^h^Population comprising more conditions than LBP, numbers refer to the general population and were not available for LBP group only.

^i^Protocol paper, no data available unless reported alongside the RCT results paper.

^j^Planned number to recruit based on protocol paper.

### Quality Assessment

Results for the 7 items of the Cochrane Risk of Bias tool for the 6 completed RCTs are reported in Table 2. Four studies had one or more items rated as unclear risk of bias [28,29,32,35], 4 studies had one item with high risk of bias [28,32,35,37], and only one study had a low risk of bias for all 7 items [33]. One study was assessed to have a high risk of incomplete data [35] because the attrition rate was extremely high. Two studies were rated as high risk of selective outcome reporting as one had no published protocol and reported the outcome with highest effect size as a primary outcome [28], and the other did not report on the primary outcome stated in the published protocol [32]. One study was assessed to have high risk of bias for other potential biases due to differences in educational level between the groups [37].

### Key Intervention Components and Theoretical Underpinning of Digital Self-Management Interventions for LBP

#### Content

The extent of descriptions of the intervention content varied across studies (Table 3), but the level of details provided was generally sparse. The content of the digital interventions can be grouped into the following categories: (1) Pain education material: all studies report educational material as part of the intervention, which included information on pain origin, mechanisms and management, epidemiology of LBP, psychological aspects (eg, role of depression and mood), diagnostics and treatment-options; (2) General well-being activities: information concerning well-being, such as meditation, relaxation, general physical activity, and sleep hygiene, was reported in 4 studies [27-29,31]; (3) Exercise advice and goals: 5 studies described exercise advice, such as recommendations and goal-setting [27,29,31-33]. Two studies included short videos of exercises [32,33]; (4) E-community: one study reported a discussion forum with peers and health professionals in addition to the educational material [33]; (5) Narratives: one study included patient stories as part of the content [28].

**Table 2:** Quality assessment: risk of bias assessment with Cochrane Risk of Bias tool (low risk of bias, unclear risk of bias, high risk of bias).

Study	Random sequence generation	Allocation concealment	Blinding		Incomplete data		Selective reporting			Other biases
			Patients/personnel	Assessor						
Chiauzzi et al [29]	Low	Unclear	Unclear	Low	Low		Unclear		Unclear	
Simon et al [35]	Low	Low	Unclear	Low	High		Low		Low	
Carpenter et al [28]	Low	Unclear	Unclear	Low	Low		High		Unclear	
Krein et al [33]	Low	Low	Low	Low	Low		Low		Low	
Irvine et al [32]	Unclear	Unclear	Unclear	Low	Low		High		Unclear	
Weymann et al [37]	Low	Low	Low	Low	Low		Low		High	

#### Theoretical Underpinnings

Four of the 9 studies reported a theoretical underpinning to their intervention development (Table 3) [28,29,32,33]; cognitive behavior theory [28,29], collaborative decision making [29], social cognitive theory [32,33], theory of planned behavior [32], and acceptance and commitment therapy [28] were reported. The following approaches were mentioned as underpinning or rationales for the intervention: mindfulness [28], person-based approach [31], and self-management principles (not specified further) [29]; and tools such as goal setting [33] and information on pain and pain etiology [32,36]. Finally, 2 studies also reported that the advice given to participants was based on treatment guidelines, either evidence-based or recommendations from Governmental Institutes [27,37]. With regard to the tailoring element of interventions, 2 of the 7 studies that reported a tailoring element to their intervention described a systematic theoretical underpinning for the tailoring: Simon et al used the Ottawa Decision Support framework [35], whereas Weymann et al used the Avoidance Endurance Model and Health-Literacy as tailoring frameworks, as described in the study protocol [30,37].

**Table 3:** Intervention components and theoretical underpinnings.

Study	Content	Theoretical underpinning of content
Chiauzzi et al [29]	Educational material: content not more specifically described Wellness activities: enhance good sleep, nutrition, stress management, exercise practices	Cognitive behavior theory Collaborative decision making
Simon et al [35]	Condition-specific information: epidemiology, etiology, diagnostics, treatment options	N/R^a^
Carpenter et al [28]	Educational chapters: all about pain, thoughts and pain, stress and relaxation, getting active Didactic material and interactive exercises Patient stories Guided relaxation and meditation exercises	Cognitive therapy, behavioral activation Acceptance and commitment therapy
Krein et al [33] Krein et al^c^[34]	Educational material: Handouts about topics (body mechanics, use of cold packs, lumbar rolls, and good posture); videos demonstrating specific strengthening and stretching exercises Pedometer data: weekly goals for steps E-community: participants to post suggestions, ask questions, and share stories	Social cognitive theory^b^
Irvine et al [32]	Education and behavioral strategies to manage and prevent pain: thirty 1-4 min videos on pain management, cognitive, and behavioral strategies; videos gain-framed messages with animated whiteboard-style coach; videos of ergonomics and exercises	Social cognitive theory Theory of planned behavior
Weymann et al [37] Dirmaier et al^c^[30]	Educational information: physiology of pain, acute versus chronic pain; “chronification”; epidemiology; psychological aspects; coping and pain management Diagnostic procedures Treatment options	N/R
Geraghty et al^c^[31]	Educational information: goal review; feedback on achievements; sessions on sleep, pain relief, flare-up, work, mood daily living. Supporting advice: managing pain; modeling expectation through patient stories; reinforcing positive behavior through automated feedback; simple instructions on back exercises/behavior	N/R
Valenzuela-Pascual et al^c^ [36]	Content not yet developed, but will be based on qualitative study including interviews with patients	N/R
Amorim et al^c^ [27]	Educational material: “make your move—sit less, be active for life!” Information on how to increase physical activity and decrease sedentary behavior Health-coaching by health care professional FitBit activity monitor/feedback device	N/R

^a^N/R: not reported.

^b^Information given in the protocol but not stated in the randomized controlled trial report.

^c^Protocol paper.

### Outcome Measures Used in Digital Self-Management Interventions for LBP

#### Primary Outcomes

A wide range of outcomes were included in the RCTs (Table 4), with a total of 16 different outcomes being reported as a “primary outcome” measure. The number of primary outcomes per study ranged from 1 to 4. The primary outcome measures covered the domains of pain-related disability, pain intensity, attitude, depression, physical activity, knowledge of LBP, markers of self-care, and participant’s assessment of change over time. Of the 6 completed trials, 4 studies [32,33,35,37] did not find a statistically significant effect on the primary outcome measures in favor of the intervention group; one study [28] reported a statistically significant effect in favor of the intervention compared with the control group on 6 of 7 subscales of their primary outcome—Survey of Pain Attitudes (SOPA)—following 3 weeks of intervention use (*F* statistic ranged from 5.1 to 44.7); while Chiauzzi et al [29] reported a favorable effect in the intervention arm but only in one of 4 primary outcomes that they measured (the Patient Global Impression Change Scale).

##### Pain-Related Disability

Pain-related disability was considered as the primary outcome in 4 of the 9 studies. The Roland-Morris Disability Questionnaire (RMDQ) was used in 2 of the 6 completed RCTs [28,33]. Carpenter et al [28] reported a significant difference in favor of the intervention group in RMDQ after 3 weeks of Web-based intervention compared with a waiting list control group (a reduction in RMDQ score of 2.8 for the intervention group compared with 0.8 for the control group; *P*=.01). Krein et al [33] similarly used the RMDQ, but observed reduced disability in chronic LBP with a 12-month, pedometer-based, Internet-supported, intervention of the same magnitude as the control group. The 3 protocols for RCT trials [27,31,36] all expected to use RMDQ as a measure of pain-related disability. The Oswestry Disability Index (ODI) was stated as the primary outcome measure in two RCT reports [29,32]. Chiauzzi et al [29] did not find a difference in ODI score between the intervention and control group after 4 weeks of access to a pain information website compared with static participant information. Irvine et al [32] did not report the trial results for ODI even though it was stated as a primary outcome in their Web-based trial registration.

#### Secondary Outcomes

A large variety of secondary outcome measures were described (Table 4 and Multimedia Appendix 2 provide a more detailed view). The outcome measures covered the following domains: pain-related disability; pain; health-related disability; depression/mood; fear of movement; pain catastrophizing; physical activity; knowledge of LBP, markers of self-care, and a range of other outcomes not held within our *a priori* defined domains. For the 3 protocols of future RCTs [27,31,36], a more consistent choice of outcomes was seen, as 2 outcomes—RMDQ and pain intensity—were planned to be measured in all 3 RCT protocols and 3 outcomes—the Tampa Scale of Kinesiophobia (TSK) [31,36], Pain Catastrophizing Scale (PCS) [31,36], and the International Physical Activity Questionnaire (IPAQ) [27,31]—were planned in 2 of the 3 RCT protocols. Below we provide an overview from the 6 included RCT reports of the treatment effects observed for the secondary outcomes that we had identified as being of interest in our systematic review protocol.

##### Pain Intensity

Pain intensity measured with either an 11-point Numerical Rating Scale (NRS) or a 100-mm Visual Analogue Scale (VAS) was reported in 3 of 6 RCT reports [28,32,33]. Only one study [32] reported that the digital intervention had a beneficial effect on pain intensity, 16 weeks post-intervention (eta-square = 0.43, *P*=.002); however, this was reported as a composite pain measure combining pain intensity, duration, and frequency.

##### Quality of Life

Health-related quality of life was reported in 2 studies using the Dartmouth Primary Care Cooperative Information Project (CO-OP) [32] and the Short-Form 12-Item questionnaire (SF-12) [34]. Only one of these studies actually reported the effect in the RCT report. Again Irvine et al used a composite outcome measure, incorporating functionality, well-being, and quality of life; however, they reported a beneficial effect of the intervention compared with the control arm (eta-square = 0.033, *P*=.001) [32].

##### Depression

Depression was reported in 3 of the 6 RCT reports [28,29,33] but only one study reported beneficial effects of the digital intervention using the Negative Mood Regulation Scale (an increase in score of 0.4 in the intervention group compared with 0.1 in the control group after 3 weeks of the intervention, *P*<.001) [28].

##### Fear Avoidance

Three studies reported fear of movement with the Fear Avoidance Belief Questionnaire (FABQ) [28,29,33], but only one reported an effect in favor of the digital intervention group [28], just for the physical activity subscale (decrease in score of 1.0 compared with an increase of 0.1 in the control group, after 3 weeks of the intervention, *P*<.001). One study used the TSK as a measure of fear avoidance; no between-group difference was reported [32].

##### Pain Catastrophizing

The PCS questionnaire was used in 2 RCT reports [28,29] but again only one study reported an effect in favor of the digital intervention compared with the waiting list control for the 3 subscales [28].

##### Physical Activity

Only one of the completed RCTs assessed physical activity outcomes and observed no difference in daily steps achieved between the control and intervention group [33].

##### Medication Use

No studies reported medication use.

##### Health Care Utilization

No studies reported details of health care utilization (eg, primary and secondary care visits, emergency department visits).

##### Health Care Costs

No studies reported on health care costs or cost-effectiveness.

##### Knowledge of LBP

Three of the RCT reports used participants’ knowledge of LBP as an outcome measure [32,35,37]. Simon et al [35] and Weymann et al [37] used the same self-developed questionnaire, but neither study found a difference between the digital intervention and control group. Irvine et al assessed knowledge using a self-developed questionnaire and reported an effect in favor of the intervention group, however, as a composite score of 3 different outcomes (self-efficacy, behavior intentions, and knowledge) [32].

##### Markers of Self-Care

In total, 14 different outcomes were identified as markers of self-care, such as the Decision Conflict Scale [35,37], Patient Activation Measure, [32] and Preparation for Decision Making Scale [35,37]. Overall, 5 of the 14 outcomes showed an effect in favor of the digital invention when compared with a control group. Of the 14 outcomes, 10 were reported in only 3 of 6 RCT reports. Of these, the studies by Simon et al [35] and Weymann et al [37] originate from the same research group, and consequently there is considerable overlap between the interventions described and outcomes assessed in both trials. Irvine et al reported an effect on 3 outcomes of self-care in favor of the digital mobile app FitBack, when compared with the control group (behavior intentions, Patient Activation Measure, and prevention helping behaviors) [32].

##### Self-Efficacy

Four different measures of self-efficacy were reported in 4 RCT reports. The Self-Efficacy for Exercise Scale was used by Carpenter et al, who found an effect on self-efficacy in favor of the digital intervention group as compared with the waiting list control [28]. Irvine et al used a self-developed self-efficacy scale in a composite outcome score and reported a difference in favor of the digital intervention, however, again reported in a composite score [32]. The two other studies reported no benefits [29,33].

#### Other Outcomes

Ten outcomes could not be classified within our *a priori* defined outcome domains. These 10 included work-related outcomes, such as the Stanford Presenteeism Scale (SPS) [32], time off work, [32] and the Work Limitations Questionnaire (WLQ) [32], and procedural and implementation outcomes, which included issues such as feasibility [31], treatment adherence [35], as well as credibility and expectations of the intervention [31]. Four outcomes were additionally placed in an “other” category: the Chronic Pain Coping Inventory (CPCI) [29]; Participants’ Global Impression of Change (PGIC) [29]; StartBack Screen Tool [31]; and the Problematic experience of Therapy Scale [31]. For these outcomes in the completed RCTs, Irvine et al reported a between-group difference favoring the digital intervention for the SPS and WLQ in a composite score [32] and Chiauzzi et al reported between-group differences favoring the digital intervention for the CPCI and PGIC outcomes [29].

### Specific Characteristics or Components of Digital Self-Management Interventions for LBP Associated With Beneficial Outcomes

Key characteristics of the digital interventions are summarized in Table 5.

#### Aim of Interventions

Eight of the 9 studies aimed to investigate the effectiveness of the digital intervention in relation to pain intensity, attitudes toward pain, or pain-related disability by comparison with a control group (usual care or a nondigital intervention; as summarized in Table 4) [27-29,32,33,35-37]. One study had its main objective to explore the feasibility of the digital intervention [31].

#### Intervention Characteristics

##### Format and Delivery

Seven of 9 studies assessed digital interventions that were accessed over the Internet and by use of a computer [28,29,31,33,35-37], and 2 studies assessed digital interventions, which were app based, but accessible from both computer and handheld devices (tablets or smartphones) [27,32].

##### Frequency, Duration of Use, and Intervention Duration

Large variation was seen in the reported frequency and duration of use of the digital interventions. Six studies reported unlimited access to the programs with no report of recommendations given regarding frequency of use [27,32,33,35-37]. Geraghty et al [31] recommended a frequency of 1 session per week; Carpenter et al [28] recommended participants complete 2 chapters of the program per week over the 3-week study period; and Chiauzzi et al [29] instructed participants to log in for sessions twice per week. In 3 studies, weekly reminders to visit the website or app were sent to participants in the intervention groups [28,32,33]. Although all studies provided participants with a recommended frequency of use, only 2 of the 9 studies reported their recommended duration of use per visit with a range of 20 min per session to 1-1.5 hours per session [28,29]. Several studies reported that they registered user data but did not give results. Intervention duration also varied greatly, with 3 RCTs lasting between 2 and 4 weeks [28,29,36], one lasting 8 weeks [32], 3 were 3-month long [31,35,37], one study was 6-month long [27], and the longest duration was reported to be of 12 months [33].

###### Interactive Elements

The interactive elements reported in the studies included (1) keeping a log or journal of use of the intervention [29,32]; (2) simulated dialogue between the user and the system, where the user’s answer(s) was (were) used to create individualized information [28,35,37]; (3) small exercises, such as quizzes, drag-and-drop questions [28]; (4) patient’s report of outcome data and receiving feedback in the form of revised goals, for example, goals for steps per day based on pedometer data [27,33] or graphs illustrating changes in pain intensity [27,32]; (5) targeted messages with information and motivational feedback from the system [27,31-33]; and (6) Web-based discussion forums with peers and health care professionals [33].

**Table 4:** Study aim, available outcomes, and main results.

Study	Aim	Primary analysis		Secondary outcomes	Main result	Control condition
		Outcomes	Measurement Times			
Chiauzzi et al [29]	Compare interactive self-management website for chronic LBP to standard text-based materials; hypothesized improved emotional management, coping, self-efficacy to manage pain, pain levels, and physical functioning	BPI (Brief Pain Inventory) ODQ (Oswestry Disability Questionnaire) DASS (Depression/Anxiety and Stress Scale) PGIC (Patient Global Impression of Change scale)	Baseline, post-intervention (4 weeks), 3 months, 6 months	PCS (Pain Catastrophizing Scale) FABQ (Fear Avoidance Belief Questionnaire)	Hypothesis not supported	Educational material: “A back pain guide” No reminder emails
Simon et al [35]	Whether insurees with depression or LBP experienced more favorable decision-related outcomes after using a Web-based tailored decision aid compared with non-tailored, static patient information	DCS (Decisional Conflict Scale)	Baseline, post-intervention, 3 months	Preparation for decision-making scale Preference for participation, knowledge Doctor facilitation Information exchange Decision regret Treatment adherence	Intervention effective in short term Follow-up data of >3 months did not suggest further effects of intervention	Same information as intervention, website, but no tailoring to the individual user
Carpenter et al [28]	Efficacy of a pilot version of a Web-based CBT (cognitive behavioral therapy) intervention for chronic LBP	SOPA (Survey of Pain Attitudes)	Baseline, 3 weeks, 6 weeks	FABQ NMR (Negative Mood Regulation scale) PCS RMDQ PSES (Pain Self-Efficacy Scale) Demographics and pain assessment questionnaire	Difference in favor of the intervention group on all SOPA subscales in the SOPA questionnaire except “medical cure”	Wait list, received no care for 3 weeks, then access to website
Krein et al [33] Krein et al [34]^a^	Whether a pedometer-based, Internet-mediated intervention would reduce pain-related disability and functional interference in chronic LBP	RMDQ (Roland-Morris Disability Questionnaire) SF-36 function scale	Baseline, 6 months, 12 months	Pain intensity (NRS, numerical rating scale) Walking (steps/day) FABQ PA (physical activity) subscale Self-efficacy 6-min walking test^b^ CES-D 100^b^(Centre for Epidemiologic Studies Depression Scale)	No between-group difference reported at any time-points	Usual care (attending Back Class) and uploading pedometer data after receiving monthly email reminders to upload; no goal-setting or feedback received; no access to website
Irvine et al [32]	Test FitBack for adults at increased risk for chronic LBP due to a recent episode of NLBP	*No primary outcome stated* ODQ stated as primary outcome in trial registration	Baseline, 8 weeks, 16 weeks	Pain: level, frequency, intensity and duration MPI (Multidimensional Pain Inventory Interference Scale) Dartmouth CO-OP Prevention-helping behaviors (self-developed) WLQ (Work Limitations Questionnaire) SPS (Stanford Presenteeism Scale) PAM (Patient Activation Measures) Knowledge Behavioral intensions Self-efficacy SOPA (modified) TSK (Tampa Scale of Kinesiophobia; modified)	*No data available for primary outcome analysis*	Usual care, emails to request completion of questionnaire
Weymann et al [37] Dirmaier et al [30]^a^	Investigate effectiveness of a Web-based, tailored, fully automated intervention for patients with type-2 diabetes or chronic LBP against a standard website with identical content without tailoring	Knowledge (post-intervention) Patient empowerment (heiQ, Health Education Impact Questionnaire; 3 months)	Baseline, post-intervention, 3 months	DCS PDMS (Preparation for Decision Making Scale)	The tailored intervention had no effect on the total study population	Same website material as intervention but not tailored; not presented in a dialogue format; no guidance through the content
Geraghty et al [31]^a^	Explore feasibility of providing an Internet intervention for patients with LBP in primary care, with and without physiotherapist telephone support (in addition to usual care), compared with usual care alone	Feasibility outcome *Number need to screen* *Recruitment rates* *Login and usage information*	Baseline, 3 months	Pain: days, duration, intensity RMDQ StartBack Screen Tool TSK PCS IPAQ (International Physical Activity Questionnaire) PEI (Patient Enablement Instrument) EQ-5D (Euro-Qol 5D) LBP related health care use Time off work CEQ (Credibility and Expectancy Questionnaire) SESE (Self-Efficacy for Exercise Scale) PETS (Problematic Experiences of Therapy Scale)	-	Usual care from their general practitioner; this may consist of education and self-management advice, including advice to stay active
Valenzuela-Pascual et al [36]^a^	Evaluate effect of a biopsychosocial Web-based, educational intervention for chronic LBP based on pain intensity compared with normal care	Pain intensity (100-mm VAS [visual analogue scale] scale)	Baseline, 2 weeks	FABQ TSK PCS RMDQ SF-36	-	No intervention; asked to return to webpage to complete questionnaire at 2 weeks
Amorim et al [27]^a^	Investigate effect of a patient-centered PA intervention supported by health coaching and technology in chronic LBP	Care-seeking Pain levels (NRS) RMDQ	Baseline, weekly during intervention, 6 months, 12 months	IPAQ *Actigraph* accelerometer GAS (Goal Attainment Scale)	-	Educational material same as intervention: “Make your move—Sit less, be active for life!”; advice to work toward increasing PA and achieving long-term goals

^a^Protocol paper, no data available.

^b^Difference between the protocol paper and RCT report.

##### Tailoring

Two of the 9 studies did not report any tailoring element to the content of their digital intervention [27,28]. Valenzuela-Pascual et al [36] did not specify the information they used for tailoring. Of the other 6 studies, all used some form of patient characteristics to inform tailoring, for example, Krein et al [33] used gender as a tailoring variable; Chiauzzi et al [29] used participant responses and characteristics (not further specified); Irvine et al [32] used job-type assessed by questionnaires; and Geraghty et al [31] used the extent to which LBP obstructed daily activities as a tailoring variable.

**Table 5:** Intervention characteristics.

Study	Mode of delivery	Recommended frequency	Recommended duration of visit	Interactive element	Tailoring	Intervention Duration Attrition rate^g^
Chiauzzi et al [29]	Website	2 times/week for 4 weeks, then unlimited	<20 min/session	Log of activities and content viewed during sessions	Yes Matched patient characteristics to educational content, articles, and interactive tools	4 week intervention period, access for 6 months Attrition rate: 6 months I^a^=67/104 C^b^=88/105
Simon et al [35]	Website *Small information units presented in combination of text and graphics*	Unlimited access but no required frequency	N/R^c^	Simulated dialogue between user and system Text or graphics varied based on needs of users	Yes Ottawa Decision Support Framework Tailoring based on ≥4 tailoring concepts, including patient characteristics and preferences	One-time use required, access for 3 months Attrition rate: Post-use I^a^=147/691 C^b^=195/652 3 months I^a^=40/691 C^b^=25/652
Carpenter et al [28]	Website *Text and graphic with audio narration* *Animation used in educational material*	Two times/week, email reminders	1-1.5 hour/log-in	Reflective and interactive exercises	No	3-week intervention period Attrition rate: 3 weeks I^a^=63/70 C^b^=68/71
Krein et al [33] Krein et al[34]^d^	Website *Graphical and written feedback* *Motivational messages* *Weekly news updates*	Unlimited access with weekly reminders to upload data	N/R^c^	Pedometer data, used to create weekly PA^e^goals and track progress Targeted messages Discussion on Web-based forum with peers and health personnel	Yes Gender^f^ Written and graphical information as targeted messages^f^	12-month intervention period Attrition rate: 12 months I^a^=102/111 C^b^=105/118
Irvine et al [32]	Web app, accessible from Internet and mobile *Gain-framed text and video messages*	Unlimited access, weekly reminders to visit app	N/R^c^	Pain and PA^e^self-monitoring tool Journal-keeping function 7- and 30-day graphs of pain	Yes Job-type assessed by questionnaires	8-week intervention period, access for 16 weeks Attrition rate: 8 weeks I^a^=192/199 C^b^=197/199
Weymann et al [37] Dirmaier et al [30]^d^	Website	Unlimited access, designed to be used in 1 sitting	N/R^c^	Simulated dialogue between user and system User-control to navigate site by replying to at least 3 options after each text passage	Yes Avoidance Endurance Model Health literacy^f^ Motivational Interviewing Tunnelled design developed	3-month intervention period Attrition rate: 3 months I^a^=96/190 C^b^=106/188
Geraghty et al [31]^d^	Website	One session/week	N/R^c^	User selects PA^e^, system generates activity goals User may navigate the content as they find best	Yes Extent of pain obstructing daily activities	3-month intervention period
Valenzuela-Pascual et al [36]^d^	Website *Changing delivery formats* *Video, 2-3D animation*	Unlimited access	N/R^c^	N/R^c^ *(content not yet developed)*	Yes *(content not yet developed)*	2-week intervention period
Amorim et al [27]^d^	App, accessed via computer or smartphone	Unlimited access, no recommendations on frequency or duration	N/R^c^	User reports PA^e^levels, pain intensity, and disability User receives encouragement based on PA^e^level	No	6-month intervention period

^a^C: control group.

^b^I: intervention group.

^c^N/R: not reported.

^d^Protocol paper, no data available.

^e^PA: physical activity.

^f^Information given in the protocol but not stated in the RCT report.

^g^Attrition rates reported as number of completed cases in relation to the total number of participants randomized to the group.

## Discussion

### Principal Findings

We have systematically searched and reviewed the literature pertaining to interactive, digital interventions for self-management of LBP. The effectiveness of interventions was mixed, with only 1 study reporting a positive effect on their primary outcome [28]. We found a large degree of heterogeneity regarding the description of intervention content and delivery, theoretical underpinnings, and outcomes reported, making comparison between interventions difficult. A comprehensive description of intervention development and use of theory has been recommended when reporting on RCTs of digital interventions [38]; however, such descriptions were either brief or completely lacking in the included studies. Participants were predominantly female, white, younger, and well educated, which renders the external validity of the identified studies as low.

Despite international recommendations for reporting core outcome domains (physical functioning, pain intensity, and health-related quality of life) in LBP studies [23], we identified 16 different primary outcome measures and a total of 52 outcomes covering a wide range of domains. Better consistency in choice of outcome measures was seen in the 3 RCT protocols of planned RCTs [27,31,36]. We expect that these trials will provide more useful information and data for future meta-analyses. Generally, the included studies were not able to demonstrate significant beneficial effects on either the primary or secondary outcomes and we were unable to identify specific characteristics of interventions to explain these findings. However, it may be that the most important factors related to whether an individual engages with a digital support tool were not taken into account, for example, low mood and additional physical comorbidities. Surprisingly, physical activity, which is considered one of the mainstays of the treatment of LBP [10], was only included as a key component in one study. Consequently, no evidence was presented to support effects on physical activity behavioral changes from digital self-management for LBP. This should be a matter for focus for future RCTs in this area. None of the studies showed any evidence of harm from interactive digital interventions. There was no evidence regarding cost-effectiveness of interactive digital interventions.

### Strengths and Limitations

This systematic review was undertaken by a team with extensive experience in conducting such reviews. We used multiple databases, and a thorough search strategy that was designed iteratively by the research team and an information specialist to account for the 3 different dimensions of the search (back pain, digital interventions, and self-management). The methodological assessment tool used in our systematic review has been specifically developed to assess the risk of bias in RCTs [26], and its constructs are in line with the recommendations of the PRISMA statement [18]. All aspects of data extraction, quality appraisal, and data analysis were carried out independently by two researchers, with a third party available for adjudication in case of disagreements.

The primary limitation of this systematic review is the sparse literature related to our objectives. Due to the sparsity and heterogeneity of the data, a formal meta-analysis was not possible. Additionally, our search was limited to studies published in English, Danish, or Norwegian, which could be construed as a limitation, although there is increasing evidence that this is not a particular problem [39]; six papers were excluded at the abstract screening stage of this review based on language. Finally, gray literature was not included; however, given the nature of this review and that there is no suggestion of publication bias, it is unlikely that this will have any impact on the results.

### Comparison With Previous Literature

To the best of our knowledge this is the first systematic review of RCTs of interactive digital interventions for self-management of LBP. However, systematic reviews of Web-based interventions for LBP (not specifically self-management) [16], nondigital self-management for LBP [13], and chronic musculoskeletal pain [40] have been published. The first review suggests that CBT-based approaches and interventions that offer Web-based support may have some effect on reducing pain-related catastrophizing and improving patient attitudes; however, study quality was relatively low and further studies were recommended [16]. Reviews of interventions targeted specifically at self-management have suggested that there is only moderate-quality evidence that self-management has small effects on pain and disability in people with LBP [13,40]. These reviews have not dismissed self-management as a treatment option for LBP, but rather suggested that further research is needed to understand the limitations of self-management and whether or how effectiveness can be increased. In addition, these reviews have suggested that future studies should extend the outcomes of interest to include aspects of self-efficacy, and also consider the impact of the duration of the intervention [13,40], increase the length of follow-up [16], and also consider the impact of such interventions on health care utilization [16]. Similar conclusions have been made in systematic reviews of digital self-management interventions in conditions like asthma [22], hypertension [19], and problematic cannabis use [41]. Tailoring digital interventions to individual patient needs has been advocated to enhance engagement [42]. Our review highlights that although 5 out of 6 of the RCT reports included some form of tailoring, there was a lack of detail on exactly what this involved and the role it played in the outcome of the RCT or in user engagement. Finally, small and very similar effects across types of interventions such as different types of exercises, manual treatment, or acupuncture for people with LBP are well recognized [43]; however, because of the enormous societal impact of LBP and LBP-related disability, these interventions may still have worthwhile effects both at the patient and population level [43,44]. In this context, digital interventions aiming to promote self-management are particularly attractive because they are easy to deliver, inexpensive, and safe.

### Study Implications

The populations within the identified studies were predominantly female, white, well-educated, and middle-aged, and thus the wider applicability of digital self-management interventions remains uncertain and therefore further investigation including a broader range of participants is merited. Seven of the 9 included studies specifically aimed to address the self-management of chronic LBP, and thus the usefulness of supporting self-management for acute LBP using digital tools remains underinvestigated; any such interventions for acute LBP would possibly require different advice and support to that offered for chronic LBP, as directed in clinical guidelines [10,11]. In addition, the absence of any health economics data was surprising and certainly needs to be addressed in future studies. There were a number of areas of reporting that were identified as deficient in the majority of studies in this systematic review. This suggests that going forward greater adherence to published guidelines that have recommended increasing focus on reporting of the technical aspects of the digital intervention as well as reporting the content of the intervention and its theoretical underpinnings [38,45] would be valuable. Finally, there is growing evidence that tailoring of digital interventions may be an important ingredient for success [42], and this will be an important issue to address in future RCTs of digital interventions aimed at promoting self-management of LBP. We are aware of at least one such study currently underway [46,47].

### Conclusions

Our review has highlighted that the published literature is extremely heterogeneous and that digital intervention studies for LBP are generally poorly described. The literature provides insufficient detail regarding target and participating populations, and intervention components, theoretical underpinnings, and the rationale for the wide variety of outcome measures used. This makes it difficult to gain a clear impression of what might work best, for whom and in what circumstances. It is clear that the existing evidence has not yet proven the wider utility of digital interventions for self-management of LBP for the population at large, a knowledge gap that future research should address by better characterizing participants and interventions in a way that would allow replication and by providing clear rationales for intervention components and outcome measure selection.

## Appendix

Multimedia Appendix 1 MEDLINE search strategy.

Multimedia Appendix 2 Overview of all outcome measures included.

## Abbreviations

BPI: Brief Pain Inventory
CBT: cognitive behavioral therapy
CDSR: Cochrane Database of Systematic Reviews
CENTRAL: Cochrane Central Register of Controlled Trials
CEQ: Credibility and Expectancy Questionnaire
CES-D-100: Centre for Epidemiologic Studies Depression Scale
CINAHL: Cumulative Index to Nursing and Allied Health Literature
CO-OP: Primary Care Cooperative Information Project
CPCI: Chronic Pain Coping Inventory
DARE: Database of Abstracts of Reviews of Effects
DASS: Depression/Anxiety and Stress Scale
DCS: Decisional Conflict Scale
DoPHER: Database of Promoting Health Effectiveness Reviews
FABQ: Fear Avoidance Belief Questionnaire
GAS: Goal Attainment Scale
heiQ: Health Education Impact Questionnaire
HTA: Health Technology Assessment
IPAQ: International Physical Activity Questionnaire
LBP: low back pain
MPI: Multidimensional Pain Inventory Interference Scale
NMR: Negative Mood Regulation scale
NRS: Numerical Rating Scale
NTNU: Norwegian University of Science and Technology
ODQ: Oswestry Disability Questionnaire
ODI: Oswestry Disability Index
PA: physical activity
PAM: Patient Activation Measures
PCS: Pain Catastrophizing Scale
PDMS: Preparation for Decision Making Scale
PEI: Patient Enablement Instrument
PETS: Problematic Experiences of Therapy Scale
PGIC: Patient Global Impression of Change
PRISMA: Preferred Reporting Items for Systematic Reviews and Meta-Analyses
PSES: Pain Self-Efficacy Scale
RCT: randomized controlled trial
RMDQ: Roland-Morris Disability Questionnaire
SD: standard deviation
SES: socioeconomic status
SOPA: Survey of Pain Attitudes
SPS: Stanford Presenteeism Scale
TSK: Tampa Scale of Kinesiophobia
TROPHI: Trials Register of Promoting Health Interventions
TSK: Tampa Scale of Kinesiophobia
VAS: visual analogue scale
WLQ: Work Limitations Questionnaire
