# Supplementary material for: Digital Support Interventions for the Self-Management of Low Back Pain: A Systematic Review
Source: J Med Internet Res. 2017 May 21;19(5):e179. doi: 10.2196/jmir.7290 (PMC5466697; doi:10.2196/jmir.7290)
Supplement: Multimedia Appendix 1 [file jmir_v19i5e179_app1.pdf]

MEDLINE search details

Ovid MEDLINE(R) 1946 to March Week 1 2016

1	exp Back pain/
2	(back pain$ or lumbago or back ache$ or backache$ or (lumbar adj2 pain$) or (spin$ adj2 pain$)).ti,ab,kw,kf.
3	1 or 2
4	Computer peripherals/ or Computer storage devices/ or Computer terminals/ or Modems/ or Microcomputers/ or Computers, handheld/ or Minicomputers/ or Attitude to computers/ or Computers/ or Computer systems/ or Medical informatics/ or Medical informatics applications/ or Educational technology/ or Audiovisual aids/ or Telecommunications/ or Multimedia/ or Computer-assisted instruction/ or User-computer interface/ or Hypermedia/ or Video games/ or Electronic health records/ or Social networking/ or exp Telemedicine/ or Mobile applications/
5	(computer$ or microcomputer$ or pc or pcs or mac or macs or internet or www or web or website$ or webpage$ or local area network$ or software or cellular phone$ or cellular telephone$ or mobile$ or cell phone$ or cell telephone$ or smartphone$ or smart-phone$ or smart-telephone$ or handset$ or hand-set$ or wireless or wire-less or wifi or wi-fi or gps or global positioning system$ or bluetooth or text messag$ or texting or sms or short messag$ or multimedia messag$ or multi-media messag$ or mms or instant messag$ or social media$ or facebook or twitter or webcast$ or webinar$ or podcast$ or wiki or wikis or app or apps or android$ or blackberr$ or apple$ or ios or iphone$ or ipad$ or s40 or symbian$ or windows or ((electronic$ or digital$ or device$) adj2 tablet$) or video$ or dvd or dvds or youtube or you tube or vimeo or online or on line or interactive or chat room$ or chatroom$ or blog$1 or web-log$1 or weblog$1 or bulletin board$ or bulletinboard$ or messageboard$ or message board$ or ehealth or e-health or mhealth or m-health or pda or pdas or personal digital or device-based or email$ or e-mail$ or electronic mail$).ti,ab,kw,kf.
6	4 or 5
7	3 and 6
8	limit 7 to yr="2000 -Current"

(computer$ or microcomputer$ or pc or pcs or mac or macs or internet or www or web or website$ or webpage$ or local area network$ or software or cellular phone$ or cellular telephone$ or mobile$ or cell phone$ or cell telephone$ or smartphone$ or smart-phone$ or smart-telephone$ or handset$ or hand-set$ or wireless or wire-less or wifi or wi-fi or gps or global positioning system$ or bluetooth or text messag$ or texting or sms or short messag$ or multimedia messag$ or multi-media messag$ or mms or instant messag$ or social media$ or facebook or twitter or webcast$ or webinar$ or podcast$ or wiki or wikis or app or apps or android$ or blackberr$ or apple$ or ios or iphone$ or ipad$ or s40 or symbian$ or windows or ((electronic$ or digital$ or device$) adj2 tablet$) or video$ or dvd or dvds or youtube or you tube or vimeo or online or on line or interactive or chat room$ or chatroom$ or blog$1 or web-log$1 or weblog$1 or bulletin board$ or bulletinboard$ or messageboard$ or message board$ or ehealth or e-health or mhealth or m-health or pda or pdas or personal digital or device-based or email$ or e-mail$ or electronic mail$).ti,ab,kw,kf.
