# Supplementary material for: Digital Support Interventions for the Self-Management of Low Back Pain: A Systematic Review
Source: J Med Internet Res. 2017 May 21;19(5):e179. doi: 10.2196/jmir.7290 (PMC5466697; doi:10.2196/jmir.7290)
Supplement: Multimedia Appendix 2 [file jmir_v19i5e179_app2.pdf]

Multimedia appendix 2: Overview of all outcome measures included

	RCT reports							RCT protocols				
	Chiauzzi et al, 2010	Simon et al, 2012	Carpenter et al, 2012	Krein et al, 2013 *Krein et al, 2010*	Irvine et al, 2015	weymann et al, 2015 *Dirmaier et al 2013*	No. times reported RCT reports	Geraghty et al, 2015	Valenzuela-pascual et al, 2015	Amorim et al, 2016	No. Times reported in RCT rotocols	No times reported in total
Pain-related disability												
- Roland-Morris Disability Questionnaire (RMDQ)			↑	←			2	x	x	x	3	5
- Oswestry Disability Questionnaire (ODQ)	←				a,b		2				0	2
Pain												
-Duration					↑ [c]		1	x			1	2
-Frequency					↑ [c]		1				0	1
-Intensity, (NRS, VAS)			←	←	↑ [c]		2	x	x	x	3	5
-Brief Pain Intensity (BPI)	←						2				0	2
-Multidimensional Pain Inventory Interference Scale (MPI)					↑ [d]		1				0	1
- Survey of pain attitudes (SOPA)			↑		↑		2				0	2
Health-related quality of life												
- Dartmouth Primary Care Cooperative Information Project (CO-OP)					↑ [d]		1				0	1
- EuroQoL-5 dimension (EQ-5D)							0	x			1	1
- Short Form-12 items (SF-12)				a,b			1				0	1
- Short-Form-36items, (SF-36 QoL subscale)							0		x		1	1
Depression/mood												
-Depression Anxiety Stress Scale (DASS)	←						1				0	1
-Centre for Epidemiologic Studies Depression Scale (CES-D 10)				a,b			1				0	1
- Negative Mood Regulation Scale (NMRS)			↑				1				0	1
Fear of movement												

- Fear-Avoidance Belief Questionnaire (FABQ)	[b]		↑	←			3		x		1	4
- Tampa Kinesiophobia Scale (TKS).					←		0	x	x		2	2
Pain catastrophizing												
- Pain catastrophising Scale (PCS)	[b]		↑				2	x	x		2	4
Physical activity												
- 6 min walking test				[a,b]								0
- International Physical activity Questionnaire (IPAQ)							0	x		x	2	2
- Pedometer				←			1			x	1	2
- Short-Form-36 (SF-36, function subscale)				←			1				0	1
Medication use												
Health-care utilisation												
Health-care cost												
Knowledge of LBP												
- Knowledge (self-developed)		←				←	2				0	2
- Knowledge (self-developed, 14 items)					↑[e]		1				0	1
Markers of self-care												
- Behaviour Intentions					↑[e]		1				0	1
- Care seeking associated with LBP							0			x	1	1
- Decision regret		←					1				0	1
- Decisional Conflict Scale (DCS)		←				←	2				0	2
- Doctor facilitation		←					1				0	1
- Enablement coping (PEI)							0	x			1	1
- Goal attainment Scale (GAS)							0			x	1	1
- Health Education Impact Questionnaire (heiQ)						←	1				0	1
- Information exchange		←					1				0	1
- LBP-health-care use questionnaire (self-developed)							0	x			1	1
- Patient Activation Measure (PAM)					↑		1				0	1
- Preparation for Decision Making Scale (PDMS)		↑				←	2				0	2
- Preparation for participation		↑					1				0	1
- Prevention helping behaviours					↑		1				0	1
Self-efficacy												

-Exercise Regularly Scale (ERS)				←			1				0	1
-Pain Self-Efficacy Questionnaire (PSEQ)	←						1				0	1
-Self-Efficacy for Exercise Scale (SESE)			↑				1	x			0	1
- 13 item, 7 point Likert scale (self-developed)					↑[e]		1				0	1
Other												
- Credibility and Expectancy Questionnaire (CEQ)							0	x			1	1
- Chronic Pain Coping Inventory (CPCI-42)	↑						1				0	1
- Feasibility outcomes							0	**x**			1	1
- Treatment adherence		←					1	x			1	2
- Participants global improvement change (PGIC)	**↑**						1				0	1
- Problematic Experience of Therapy Scale (PETS)							0	x			1	1
- Risk of sustained disability (StartBack Screen Tool (SBST))							0	x			1	1
- Stanford Presentiism Scale (SPS)					↑[f]		1				0	1
- Time off work							0	x			1	1
- Work Limitations Questionnaire (WLQ)					↑[f]		1				0	1

↑: indicates a significant improvement in favour of the intervention ←: indicates no difference between groups, ↓: indicates a significant improvement in favour of the control group; **bold red** represents the primary outcomes of the study.
x: indicates the outcomes stated in protocol papers to be assessed; ; **bold red** represents the proposed primary outcomes of the study

[a] outcome reported in the trial registration or protocol paper but not reported in the published RCT report
[b] the paper stated to have assessed the outcome but no statistics concerning the difference between groups were reported
[c] pain measures were collapsed an reported as one
[d] two outcomes were collapsed and reported as one
[e] three outcomes were collapsed and reported as one
[f] two outcomes were collapsed and reported as one

Note: if several analyses were performed in one study then the analysis that included the most participants was used in this overview. If analysis was performed at several time points then the analysis at the time of the primary endpoint was used, if given, it this was not given, then the time point with the longest follow-up period was used.
